# Micronutrient balance and brain function: neuropsychological, metabolic, and clinical interactions

**DOI:** 10.3389/fmolb.2026.1748487

**Published:** 2026-04-01

**Authors:** Valeria López-Sebastiani, Karen V. Quiroz-Cornejo, Martha Paola Arellano-Salazar, Fernando Monje-Bolivar, Víctor J. Samillan

**Affiliations:** School of Nutrition and Dietetics, Universidad Le Cordon Bleu, Lima, Peru

**Keywords:** brain development, brain function, micronutrient, oxidative balance, vitamins

## Abstract

Micronutrients, encompassing both vitamins and trace elements, play a central role in brain development, metabolic homeostasis, and cognitive performance from early life through old age. Rather than acting solely as enzyme cofactors or antioxidants, many of these nutrients influence transcriptional programs, shape synaptic signaling, and participate in neuroimmune and neuroendocrine crosstalk, and observational, interventional, and experimental studies now show that both deficiency and excess of specific micronutrients are linked to changes in memory, mood, attention, and executive function, as well as increased vulnerability to neurodevelopmental, neurodegenerative, and affective disorders. In this narrative review, we summarize mechanistic and clinical evidence on the contribution of key vitamins (A, D, E, C, B-complex, and choline) and minerals (iron, zinc, iodine, magnesium, selenium, copper, among others) to brain function, highlighting shared molecular pathways related to neuroplasticity, synaptic integrity, energy metabolism, oxidative balance, and neuroinflammation, and examining how micronutrient status interacts with aging, genetic variation, and lifestyle factors such as physical activity and diet. We also discuss the neurological and neuropsychological consequences of micronutrient imbalance and the potential of targeted, personalized nutritional strategies for brain health promotion and disease prevention in vulnerable groups and across diverse settings, arguing that clarifying these interactions provides a framework for integrating micronutrient assessment into multidomain approaches to preserve cognitive function and mental wellbeing throughout the lifespan.

## Introduction

The human brain accounts for only about 2% of the total body mass yet consumes close to 20% of daily energy expenditure, reflecting its high metabolic demand and continuous electrical activity (Bourre, 2006; Prado and Dewey, 2014; Gómez-Pinilla, 2008). This energetic asymmetry makes brain function particularly sensitive to nutritional status, and especially to the availability of micronutrients that modulate enzymatic reactions, redox balance, neurotransmission, and gene expression (Bourre, 2006; Gómez-Pinilla, 2008; Gombart et al., 2020; Ames, 2018; DeLuca et al., 2013; Brigelius-Flohé and Traber, 1999; Harrison and May, 2009; Kennedy, 2016; Verdin, 2015; Zimmermann, 2009). While macronutrients provide the energetic substrate for neuronal activity, vitamins and minerals required in much smaller amounts act as indispensable regulators of cerebral homeostasis (Bourre, 2006; Prado and Dewey, 2014; Gómez-Pinilla, 2008; Ames, 2018; Zimmermann, 2009; Zhang et al., 2023; Balion et al., 2012; Hisada et al., 2022).

Despite substantial improvements in global food availability over recent decades, micronutrient deficiencies remain common in both low- and middle-income countries and highly industrialized settings (Derbyshire, 2018; Bailey et al., 2015; Mayo-Wilson et al., 2011; He et al., 2014; Bjelakovic et al., 2007). Suboptimal intakes of vitamins A, D, and E, folate, and several minerals, most notably iron, zinc, and iodine have been documented across age groups, often in the absence of overt clinical signs of deficiency (Zimmermann, 2009; Hisada et al., 2022; Derbyshire, 2018; Bailey et al., 2015; Mayo-Wilson et al., 2011; He et al., 2014; Bjelakovic et al., 2007; Troesch et al., 2015). Neurological consequences of these imbalances may nonetheless emerge much earlier, presenting as subtle changes in cognition, mood, or behavior long before classical deficiency syndromes are recognized (Prado and Dewey, 2014; Gómez-Pinilla, 2008; Ames, 2018; Kennedy, 2016; Hisada et al., 2022; Bailey et al., 2015; He et al., 2014; Troesch et al., 2015; Mikkelsen et al., 2017; Etgen et al., 2012; Presse et al., 2008; Travica et al., 2017; Smith et al., 2010; Ford and Almeida, 2019).

During critical periods of neurodevelopment, an inadequate supply of micronutrients can disrupt key processes such as neuronal proliferation and migration, myelination, and synapse formation, leaving functional sequelae that persist into later life (Prado and Dewey, 2014; Harrison and May, 2009; Verdin, 2015; Zimmermann, 2009; Hisada et al., 2022; Maden, 2007; Velasco et al., 2018; Bougma et al., 2013; Lozoff and Georgieff, 2006; Beard, 2003). In adulthood and aging, both deficiency and excess of specific micronutrients may accelerate cognitive decline, increase susceptibility to mood disorders, and contribute to the pathophysiology of neurodegenerative diseases (Gómez-Pinilla, 2008; Ames, 2018; Brigelius-Flohé and Traber, 1999; Zimmermann, 2009; Zhang et al., 2023; Balion et al., 2012; Bjelakovic et al., 2007; Etgen et al., 2012; Travica et al., 2017; Smith et al., 2010; Ford and Almeida, 2019; Lozoff and Georgieff, 2006; Beard, 2003; Lane et al., 2018; Sano et al., 1997; Lloret et al., 2021; Bozonet and Carr, 2019; Hare and Double, 2016; Das et al., 2021; Li et al., 2017; Barbagallo et al., 2021; Cardoso et al., 2015; Solfrizzi et al., 2011; Wu and Sun, 2017; Solfrizzi et al., 2017; Livingston et al., 2020; Wang et al., 2022).In Contasrt, in midlife and older age, micronutrient deficiencies more often arise from hidden hunger, chronic disease, medication effects, or age-related change such as reduced cutaneous vitamin D synthesis, and their influence is typically expressed as acceleration or attenuation of cognitive decline, mood symptoms, and neurodegenerative processes rather than gross developmental abnormalities (Verdin, 2015; Mikkelsen et al., 2016; Roberts et al., 2019).

The magnitude of these effects is usually shaped by genetic background, inflammatory status, hormonal milieu, and lifestyle factors such as physical activity, dietary pattern, and body composition (Ames, 2018; DeLuca et al., 2013; Harrison and May, 2009; Kennedy, 2016; Verdin, 2015; Lloret et al., 2021; Li et al., 2017; Barbagallo et al., 2021; Cardoso et al., 2015; Livingston et al., 2020; Jia et al., 2019; Gasperi et al., 2019; Cater et al., 2024; Vinceti et al., 2018; Matusheski et al., 2021; Cortés-Albornoz et al., 2021; Bekdash, 2024; Badaeva et al., 2023), Importantly, the impact of the micronutrients imbalance in brain function is highly time dependent, with deficient during the first 1,000 days of life, often producing qualitative different and more persistent effects than similar deficits arising in adulthood, wich tend to modulate trajectories of cognitive aging rather than basic neurodevelopmental arquiteture (Prado and Dewey, 2014; Black et al., 2017; Georgieff, 2020; Cusick and Georgieff, 2016; Nyaradi et al., 2013). In this early first 1,000 days window, most of the available evidence comes from studies of maternal diet, breastfeeding, complementary feeding or micronutrient fortification rather than single nutrient supplement trials, and the primary outcomes relate to neurodevelopmental milestones, school readiness, and long-term cognitive potential (Bourre, 2006; Nyaradi et al., 2013; Best et al., 2010; Grantham-McGregor et al., 2007). Against this backdrop, the present review aims to bring together mechanistic, neuropsychological, and clinical evidence on the role of micronutrients in brain function across the life course (Bourre, 2006; Gómez-Pinilla, 2008; Ames, 2018; DeLuca et al., 2013; Brigelius-Flohé and Traber, 1999; Harrison and May, 2009; Kennedy, 2016; Verdin, 2015; Zimmermann, 2009; Zhang et al., 2023; Balion et al., 2012; Etgen et al., 2012; Smith et al., 2010; Ford and Almeida, 2019; Lozoff and Georgieff, 2006; Beard, 2003; Sano et al., 1997; Lloret et al., 2021; Hare and Double, 2016; Das et al., 2021; Li et al., 2017; Barbagallo et al., 2021; Cardoso et al., 2015; Solfrizzi et al., 2011; Wu and Sun, 2017; Solfrizzi et al., 2017; Livingston et al., 2020; Wang et al., 2022; Gasperi et al., 2019; Cater et al., 2024; Vinceti et al., 2018; Badaeva et al., 2023). Rather than offering an isolated description of each vitamin or mineral, we focus on convergent pathways through which micronutrients influence neuroplasticity, metabolic efficiency, and neuronal resilience, and we examine how these pathways translate into opportunities for prevention and management of neurocognitive disorders in different life stages and epidemiological contexts (Gómez-Pinilla, 2008; Ames, 2018; DeLuca et al., 2013; Brigelius-Flohé and Traber, 1999; Harrison and May, 2009; Kennedy, 2016; Verdin, 2015; Zimmermann, 2009; Zhang et al., 2023; Balion et al., 2012; Troesch et al., 2015; Etgen et al., 2012; Smith et al., 2010; Ford and Almeida, 2019; Maden, 2007; Lozoff and Georgieff, 2006; Beard, 2003; Sano et al., 1997; Lloret et al., 2021; Hare and Double, 2016; Das et al., 2021; Li et al., 2017; Barbagallo et al., 2021; Cardoso et al., 2015; Solfrizzi et al., 2011; Wu and Sun, 2017; Solfrizzi et al., 2017; Livingston et al., 2020; Wang et al., 2022; Gasperi et al., 2019; Cater et al., 2024; Vinceti et al., 2018; Bekdash, 2024; Badaeva et al., 2023; Olson and Mello, 2010) Table 1.

**TABLE 1:** Key micronutrients involved in brain function: mechanisms, brain targets, and clinical relevance.

Micronutrient	Principal neurobiological mechanisms	Predominant brain regions/Processes	Clinical relevance and major modifiers	Key references
Vitamin D	VDR-mediated transcription; modulation of CREB phosphorylation and BDNF–TrkB signaling; regulation of oxidative defense and mitochondrial function; tuning of glutamatergic/GABAergic balance; microglial and astroglial immunoregulation	Hippocampus, prefrontal cortex, large-scale cortical–subcortical networks; neuroimmune and neurovascular interfaces	Low circulating 25(OH)D consistently associated with cognitive decline, depressive symptoms, and increased dementia risk; supplementation in deficient older adults yields modest, domain-specific benefits; effects modified by age, adiposity, inflammation, genotype, and sun exposure	(DeLuca et al., 2013; Balion et al., 2012; Etgen et al., 2012; Mikkelsen et al., 2016; Black et al., 2017)
Zinc	Vesicular zinc signaling at glutamatergic synapses; modulation of NMDA and AMPA receptors; regulation of long-term potentiation; zinc-finger transcription factor activity; redox and metallostatic control	Hippocampus, association cortex, glutamatergic synapses; neurogenesis niches	Both deficiency and excess linked to cognitive impairment and mood disorders; dysregulation implicated in amyloid aggregation and synaptic loss; low circulating zinc predicts incident dementia; effects shaped by diet, age, and metabolic status	(Gómez-Pinilla, 2008; Zhang et al., 2023; Li et al., 2017; Solfrizzi et al., 2011; Livingston et al., 2020))
Iron	Cofactor for myelination, monoamine synthesis, and mitochondrial respiration; involvement in DNA synthesis and neurotransmitter cycling; driver of Fenton chemistry and oxidative stress when unbuffered	Basal ganglia, hippocampus, cortex, white-matter tracts	Early-life deficiency impairs cognition and socio-emotional development; age- or disease-related iron accumulation associated with poorer memory and global cognition and with Alzheimer’s and Parkinson’s pathology; APOE and iron-handling genes modify risk	((Lozoff and Georgieff, 2006; Beard, 2003; Lane et al., 2018; Hare and Double, 2016; Das et al., 2021)
Iodine	Essential for thyroid hormone synthesis; thyroid-dependent regulation of neuronal proliferation, migration, differentiation, and myelination	Fetal and early-life cortex and cerebellum; critical neurodevelopmental windows	Prenatal and early-childhood deficiency causes irreversible IQ loss and executive dysfunction; mild deficiency and excess both associated with subtler cognitive changes; iodization policies must avoid both under- and over-correction	(Zimmermann, 2009; Hisada et al., 2022; Velasco et al., 2018; Bougma et al., 2013; Diachenko et al., 2024; AlBlooshi, 2025)
B-Group Vitamins (B6, B9, B12)	One-carbon metabolism; regulation of homocysteine; DNA and histone methylation; synthesis of monoamine neurotransmitters; support of myelin maintenance	Global neuronal and glial metabolism; myelinated tracts; vascular–neural interface	Low status associated with elevated homocysteine, brain atrophy, cognitive decline, and depression; B-vitamin supplementation slows brain atrophy and benefits cognition in individuals with high homocysteine; response influenced by MTHFR and related polymorphisms	(Kennedy, 2016; Smith et al., 2010; Ford and Almeida, 2019; Wang et al., 2022; Cortés-Albornoz et al., 2021; Kaye et al., 2025a)
Vitamins C and E	Vitamin C: antioxidant and cofactor for monoamine synthesis; support of neurogenesis and SVCT2-mediated uptake. Vitamin E: lipid peroxidation barrier; protection of PUFA-rich synaptic membranes; modulation of redox-sensitive signaling	High-metabolic regions (hippocampus, amygdala, cortex); synaptic membranes	Lower plasma or brain levels frequently observed in neurodegenerative diseases; adequate status associated with better cognitive performance; high-dose vitamin E slows functional decline in some Alzheimer’s trials but may increase mortality if excessive; combined antioxidant strategies appear more effective than single-nutrient approaches	(Harrison and May, 2009; Cusick and Georgieff, 2016; Nyaradi et al., 2013; Best et al., 2010; Grantham-McGregor et al., 2007; daCunha Germano et al., 2023; La Torre et al., 2023; Icer et al., 2021)
Magnesium	Voltage-dependent NMDA receptor blockade; modulation of calcium influx; support of mitochondrial stability; regulation of CREB phosphorylation and synaptic plasticity	Cortex and hippocampus; glutamatergic and stress-related circuits	Inadequate intake associated with anxiety, sleep disturbances, insulin resistance, and cognitive dysfunction; magnesium-L-threonate and other forms improve synaptic density and learning in animal models, with emerging but still limited human evidence	(Barbagallo et al., 2021; Cater et al., 2024)
Selenium	Selenoprotein-mediated antioxidant defense; regulation of thyroid hormone metabolism; modulation of redox-sensitive pathways and mitochondrial function	Widespread, with enrichment in regions vulnerable to oxidative stress; neuroendocrine axis	Both low and high selenium exposure linked to cognitive impairment and metabolic risk; optimal range appears narrow; genetic and environmental factors (soil content, diet) modulate status and response to supplementation	(Zimmermann, 2009; Cardoso et al., 2015; Gasperi et al., 2019; AlBlooshi, 2025)

## Micronutrients and neurobiological regulation of brain function

### Fat-soluble vitamins and neural plasticity

Fat-soluble vitamins tend to accumulate in tissues and act directly on gene expression programs, so their effects on the nervous system are often longer-lasting than those of many other nutrients (Bourre, 2006; Gómez-Pinilla, 2008; Ames, 2018; Brigelius-Flohé and Traber, 1999; Zimmermann, 2009; Maden, 2007; Olson and Mello, 2010; Hirota, 2023). Vitamin A, through its active metabolite retinoic acid, regulates neuronal differentiation and synaptic remodelling by activating nuclear receptors that are prominently expressed in the hippocampus and prefrontal cortex (Maden, 2007; Olson and Mello, 2010). Retinoid signalling modulates long-term potentiation in part by altering NMDA receptor expression and synaptic protein synthesis, linking vitamin A availability to learning and memory processes in both animal models and humans (Harrison and May, 2009; Verdin, 2015; Zimmermann, 2009; Maden, 2007; Olson and Mello, 2010). Consistent with this, disturbances in retinoid pathways have been associated with subtle cognitive deficits and affective symptoms in older adults, although the clinical evidence in this area remains less robust than for some other micronutrients (Harrison and May, 2009; Travica et al., 2017; Smith et al., 2010; Ford and Almeida, 2019; Maden, 2007; Olson and Mello, 2010).

Vitamin D is perhaps the most frequently cited example of a micronutrient with pleiotropic neurobiological actions, supported by both experimental and clinical data (Ames, 2018; DeLuca et al., 2013; Zimmermann, 2009; Balion et al., 2012; Etgen et al., 2012; Mirarchi et al., 2023). Beyond its classical role in calcium homeostasis, active vitamin D interacts with specific receptors (VDRs) in neurons, astrocytes, and microglia, thereby regulating gene networks involved in antioxidant defense, mitochondrial function, angiogenesis, and neurotrophic support (DeLuca et al., 2013; Verdin, 2015; Zimmermann, 2009; Balion et al., 2012; Etgen et al., 2012; Mirarchi et al., 2023). Among the best-characterized pathways are the modulation of CREB phosphorylation and the enhancement of BDNF–TrkB signaling, both of which are central to synaptic plasticity and neuronal survival (DeLuca et al., 2013; Kennedy, 2016; Verdin, 2015; Balion et al., 2012; Etgen et al., 2012; Mirarchi et al., 2023). In parallel, meta-analyses and longitudinal studies have shown that low circulating 25-hydroxyvitamin D concentrations are associated with a higher risk of cognitive decline, dementia, and depressive symptoms in older adults, whereas supplementation in deficient individuals appears to confer modest but consistent benefits in selected cognitive domains (Zimmermann, 2009; Balion et al., 2012; Etgen et al., 2012; Travica et al., 2017; Smith et al., 2010; Ford and Almeida, 2019; Jia et al., 2019; Mirarchi et al., 2023; Muhamad et al., 2023), these effects are not uniform across populations and are likely to depend on baseline status, inflammatory context, genetic background, and co-interventions such as structured exercise (DeLuca et al., 2013; Kennedy, 2016; Balion et al., 2012; Etgen et al., 2012; Livingston et al., 2020; Matusheski et al., 2021; Cortés-Albornoz et al., 2021; Bekdash, 2024; Badaeva et al., 2023Badaeva et al., 2023).

Vitamin E, particularly α-tocopherol, plays a key role in protecting neuronal membranes from lipid peroxidation, which is especially relevant in a tissue as rich in polyunsaturated fatty acids as the brain (Bourre, 2006; Gómez-Pinilla, 2008; Ames, 2018; Brigelius-Flohé and Traber, 1999; Zimmermann, 2009; Sano et al., 1997; Lloret et al., 2021; Cardoso et al., 2015). This antioxidant action helps preserve synaptic integrity and signal transmission and has been linked to slower functional decline in certain neurodegenerative conditions, especially when combined with other therapeutic strategies (Brigelius-Flohé and Traber, 1999; Sano et al., 1997; Solfrizzi et al., 2011; Wu and Sun, 2017; Solfrizzi et al., 2017; daCunha Germano et al., 2023; La Torre et al., 2023; Icer et al., 2021; MaretG, 2024). Classic clinical trials in Alzheimer’s disease have shown that high doses of vitamin E can slow functional deterioration in some patient subgroups, but they have also raised concerns about increased mortality with excessive supplementation, underscoring the importance of avoiding both deficiency and excess (Zhang et al., 2023; Sano et al., 1997; Lloret et al., 2021; MaretG, 2024; Kaye et al., 2025a).

Vitamin K, traditionally associated with coagulation, has recently emerged as a modulator of sphingolipid homeostasis and neuronal survival (Hirota, 2023; Popa et al., 2021; Emekli-Alturfan and Alturfan, 2023). It influences pathways related to microglial activation, neurovascular inflammation, and amyloid peptide clearance, and may therefore exert context-dependent neuroprotective effects, particularly in the early stages of aging (Hirota, 2023; Emekli-Alturfan and Alturfan, 2023; Diachenko et al., 2024). Observational studies have reported lower vitamin K intakes in older adults with incipient cognitive impairment, although intervention trials are still lacking to define optimal dosing and to identify which patient profiles might benefit most from targeted vitamin K strategies (Solfrizzi et al., 2011; Wu and Sun, 2017; Solfrizzi et al., 2017; Popa et al., 2021; Emekli-Alturfan and Alturfan, 2023; AlBlooshi, 2025).

### Water-soluble vitamins and neurotransmitter metabolism

Water-soluble vitamins form an interconnected metabolic network that is closely tied to neurotransmitter synthesis, redox regulation, and one-carbon metabolism (Harrison and May, 2009; Kennedy, 2016; Verdin, 2015; Smith et al., 2010; Ford and Almeida, 2019; Wang et al., 2022). Vitamin C is highly concentrated in brain regions involved in emotion and memory, such as the hippocampus and amygdala, where it is actively transported via SVCT2 (Harrison and May, 2009; Salazar et al., 2023; Han et al., 2021). Beyond acting as a first-line antioxidant, vitamin C serves as a cofactor for monoamine synthesis and contributes to synaptic maturation and protection against oxidative damage induced by excitotoxicity or inflammation (Travica et al., 2017; Bozonet and Carr, 2019; Figueroa-Méndez and Rivas-Arancibia, 2015; Zylinska et al., 2023). Experimental models further suggest that vitamin C availability influences neurogenesis and gliogenesis, reinforcing the view that its role extends well beyond that of a simple radical-scavenging “shield” (Salazar et al., 2023; Han et al., 2021; Zylinska et al., 2023).

Evidence for micronutrient effects on adult hippocampal neurogenesis remains more limited and heterogeneous than for early life neurodevelopment, and most data still come from animal and preclinical models rather than well phenotyped human cohorts (Kennedy, 2016; Verdin, 2015; Mikkelsen et al., 2016; O’Leary and Samman, 2010).

In human studies, adequate plasma levels have been consistently associated with better performance in domains such as memory, attention, and processing speed, particularly in older adults or individuals exposed to high oxidative stress (Travica et al., 2017; Bozonet and Carr, 2019; Solfrizzi et al., 2011; Wu and Sun, 2017; Solfrizzi et al., 2017; Figueroa-Méndez and Rivas-Arancibia, 2015).

The B-vitamin group provides a dense network of cofactors that support neuronal integrity and synaptic function (Gómez-Pinilla, 2008; Ames, 2018; Kennedy, 2016; Verdin, 2015; Zhang et al., 2023; Smith et al., 2010; Ford and Almeida, 2019; Wang et al., 2022; Badaeva et al., 2023). Vitamins B6, B9 (folate), and B12 are essential for homocysteine metabolism and for methylation reactions required for DNA synthesis, myelin maintenance, and the production of neurotransmitters such as serotonin, dopamine, and noradrenaline (Kennedy, 2016; Verdin, 2015; Smith et al., 2010; Ford and Almeida, 2019; El-Mezayen et al., 2022). Chronic elevations of homocysteine often arising from insufficient intake of these vitamins or from genetic variants in folate-related pathways, have been robustly linked to accelerated brain atrophy, cognitive decline, depression, and increased risk of neurodegenerative disease (Kennedy, 2016; Smith et al., 2010; Ford and Almeida, 2019; Solfrizzi et al., 2011; Wu and Sun, 2017; Solfrizzi et al., 2017; Livingston et al., 2020; Wang et al., 2022; El-Mezayen et al., 2022). Clinical trials in individuals with mild cognitive impairment indicate that combined supplementation with B6, B9, and B12 can slow the rate of brain atrophy and improve selected cognitive domains, especially when baseline homocysteine levels are high (Smith et al., 2010; Ford and Almeida, 2019; Wang et al., 2022; Badaeva et al., 2023). In this context, polymorphisms such as MTHFR C677T modulate both vulnerability to hyperhomocysteinemic damage and responsiveness to supplementation, strengthening the case for genotype-informed strategies in high-risk subgroups (Kennedy, 2016; Smith et al., 2010; Lloret et al., 2021; El-Mezayen et al., 2022).

Niacin (vitamin B3) links micronutrient status directly to cerebral energy metabolism, as it is a precursor of NAD^+^ and NADP^+^, which are central to mitochondrial function and the cellular response to metabolic stress (Gómez-Pinilla, 2008; Ames, 2018; Kennedy, 2016; Verdin, 2015; Zhang et al., 2023; Gasperi et al., 2019; Cater et al., 2024). Because the brain depends heavily on efficient oxidative metabolism to sustain synaptic transmission and plasticity, disturbances in NAD^+^ availability can compromise neuronal resilience and accelerate brain aging (Verdin, 2015; Gasperi et al., 2019; Cater et al., 2024). Recent reviews have proposed that altered NAD^+^ dynamics represent a common feature across several neurodegenerative disorders, positioning niacin as a dependent pathway including sirtuin activation as potential therapeutic targets to mitigate synaptic damage and enhance cognitive reserve (Kennedy, 2016; Verdin, 2015; Zhang et al., 2023; Mikkelsen et al., 2017; Solfrizzi et al., 2011; Wu and Sun, 2017; Solfrizzi et al., 2017; Gasperi et al., 2019). In line with this, intervention studies using NAD^+^ precursors in animal models show improvements in brain bioenergetics and behavioral outcomes, although human trials remain limited and heterogeneous in design and results (Verdin, 2015; Gasperi et al., 2019; Matusheski et al., 2021; Bekdash, 2024; Muhamad et al., 2023; Stach et al., 2021; Liwinski and Lang, 2023).

### Minerals, synaptic signaling, and brain vulnerability

Minerals in the brain act both as structural components and as direct modulators of neuronal signaling (Bourre, 2006; Gómez-Pinilla, 2008; Ames, 2018; Zhang et al., 2023; Lozoff and Georgieff, 2006; Li et al., 2017; Cardoso et al., 2015; Vinceti et al., 2018). Iodine is a classic example, as it is indispensable for thyroid hormone synthesis; these hormones, in turn, regulate neuronal migration, differentiation, and myelination during early development (Zimmermann, 2009; Bougma et al., 2013; Pinto et al., 2020). The action of thyroid hormones shows regional specificity within the central nervous system, which helps explain why iodine deficiency affects particular cognitive domains and developmental stages, even when deficiency is only mild to moderate (Pinto et al., 2020; Bath, 2024). Research involving school-aged children and pregnant women consistently shows a sobering pattern: whether iodine levels fall short or climb too high, cognitive performance suffers, manifested in lower intelligence scores, reduced attentional capacity, and diminished academic gains. These findings underscore why population-level iodine strategies must be carefully calibrated to fit local food traditions and consumption patterns (Zimmermann, 2009; Hisada et al., 2022; Velasco et al., 2018; Bougma et al., 2013; Pinto et al., 2020; Bath, 2024).

The story of iron in the brain reveals a fundamental principle: more is not always better (Zhang et al., 2023; Lozoff and Georgieff, 2006; Beard, 2003; Lane et al., 2018; Hare and Double, 2016; Das et al., 2021). During early childhood, iron plays essential roles, enabling the fatty sheaths around nerve fibers to form, supporting the synthesis of signaling molecules, and powering cellular energy factories. When iron falls short, the consequences can be surprisingly persistent, including lasting weaknesses in attention, mental processing speed, and how children relate emotionally to others (Prado and Dewey, 2014; Lozoff and Georgieff, 2006; Beard, 2003). Yet when iron begins to accumulate excessively in particular brain structures, the deep nuclei controlling movement, the memory center, or the thinking cortex, the picture darkens dramatically. Excess iron fuels the production of damaging free radicals, prompts protein clumping, and compromises cellular power generation, abnormalities recognized in Alzheimer’s and Parkinson’s patients (Ames, 2018; Zhang et al., 2023; Beard, 2003; Lane et al., 2018; Hare and Double, 2016; Das et al., 2021; Solfrizzi et al., 2011; Wu and Sun, 2017). Interestingly, how iron accumulates and whether interventions to remove or redistribute it will work both appear to depend on our genetic makeup, variations in genes like APOE and those governing iron transport seem to tip the scales toward either protection or vulnerability (Zhang et al., 2023; Lane et al., 2018; Hare and Double, 2016; Das et al., 2021; Livingston et al., 2020).

Zinc occupies a unique niche as a chemical messenger within brain circuits that use glutamate (Gómez-Pinilla, 2008; Ames, 2018; Zhang et al., 2023; Li et al., 2017). Released strategically from nerve endings in an activity-triggered manner, it fine-tunes the function of key receptor proteins and helps solidify long-term memory formation (Das et al., 2021; Li et al., 2017). Beyond defending against oxidative damage, zinc also orchestrates the activity of zinc-finger proteins, molecular switches that control genes central to memory formation and the brain’s stress-coping machinery (Li et al., 2017; Solfrizzi et al., 2011; Badaeva et al., 2023). Disruptions to zinc balance have emerged as culprits in cognitive slowing, emotional disturbances, and brain degeneration. Emerging epidemiological data paint a clear dose-dependent picture: people with lower blood zinc face substantially higher odds of developing dementia (Zhang et al., 2023; Li et al., 2017; Solfrizzi et al., 2011; Wu and Sun, 2017). Encouragingly, experimental work in animals and early human studies hint that boosting zinc may sharpen thinking and optimize certain metabolic pathways in the aging or obese brain, though large-scale, rigorous clinical trials remain sparse (Li et al., 2017; Solfrizzi et al., 2011; Livingston et al., 2020).

Other minerals including magnesium, selenium, copper, and calcium, contribute to the regulation of neuronal excitability, antioxidant defenses, and multiple intracellular signaling cascades (Gombart et al., 2020; Li et al., 2017; Barbagallo et al., 2021; Cardoso et al., 2015; Vinceti et al., 2018; Matusheski et al., 2021; Bekdash, 2024). Magnesium modulates NMDA receptor gating and stabilizes mitochondrial function, and its deficiency has been associated with excitotoxicity, neuroinflammation, and mood disturbances in experimental and observational studies (Barbagallo et al., 2021; Matusheski et al., 2021). Selenium, via selenoproteins, protects against oxidative damage, participates in thyroid hormone metabolism, and has been implicated in the pathophysiology of Alzheimer’s and Parkinson’s disease, with emerging causal signals in Mendelian randomization analyses (Zimmermann, 2009; Cardoso et al., 2015; Vinceti et al., 2018; Bath, 2024). In the case of copper and calcium, both deficiency and excess can disturb redox balance and synaptic signaling; dyshomeostasis of these ions has been described in several neurodegenerative conditions, reinforcing the notion that mineral homeostasis must be maintained within relatively narrow ranges to preserve neural function (Zhang et al., 2023; Das et al., 2021; Li et al., 2017; Solfrizzi et al., 2011) Table 2.

**TABLE 2:** Associations between micronutrient imbalance and neurological outcomes: mechanistic and clinical considerations.

Micronutrient imbalance	Neurological outcomes	Mechanistic considerations	Key references
Low Vitamin D	Cognitive decline, depressive symptoms, increased risk of dementia	Reduced VDR-mediated signaling in hippocampal and cortical neurons; impaired CREB–BDNF pathways; increased neuroinflammation and mitochondrial dysfunction; altered glutamatergic/GABAergic neurotransmission	(DeLuca et al., 2013; Balion et al., 2012; Etgen et al., 2012; Mikkelsen et al., 2016; Black et al., 2017)
Low Zinc	Memory impairment, executive dysfunction, mood and anxiety disorders; increased incidence of dementia	Disrupted vesicular zinc release at glutamatergic synapses; reduced modulation of NMDA and AMPA receptors; impaired long-term potentiation and synaptic plasticity; altered zinc-finger transcriptional regulation and redox homeostasis	(Gómez-Pinilla, 2008; Zhang et al., 2023; Li et al., 2017; Solfrizzi et al., 2011; Livingston et al., 2020)
Elevated Brain Iron (Regional Overload)	Alzheimer’s disease, Parkinson’s disease, accelerated age-related cognitive decline	Increased reactive oxygen species generation and lipid peroxidation; induction of ferroptosis; facilitation of amyloid-β and α-synuclein aggregation; mitochondrial damage in basal ganglia, hippocampus, and cortex	(Lozoff and Georgieff, 2006; Beard, 2003; Lane et al., 2018; Hare and Double, 2016; Das et al., 2021)
Iodine Deficiency (Prenatal and Early Life)	Global cognitive impairment, reduced intelligence quotient, poorer school performance	Thyroid hormone deficiency during critical neurodevelopmental windows; disrupted cortical and cerebellar maturation; impaired neuronal migration, differentiation, and myelination	(Zimmermann, 2009; Hisada et al., 2022; Velasco et al., 2018; Bougma et al., 2013; Diachenko et al., 2024; AlBlooshi, 2025)
Deficiency of B-Group Vitamins (B6, B9, B12)	Cognitive impairment, brain atrophy, depressive symptoms, vascular cognitive impairment	Hyperhomocysteinemia; impaired one-carbon metabolism; reduced DNA and histone methylation; compromised monoamine synthesis and myelin maintenance	(Kennedy, 2016; Smith et al., 2010; Ford and Almeida, 2019; Wang et al., 2022; Cortés-Albornoz et al., 2021; Kaye et al., 2025a)

### Clinical and translational implications

In recent years, there has been growing support for integrating micronutrient assessment into routine neurological and neuropsychological evaluation, at least in selected high-risk groups (Ames, 2018; Zimmermann, 2009; Zhang et al., 2023; Balion et al., 2012; Sano et al., 1997; Das et al., 2021; Solfrizzi et al., 2011; Wu and Sun, 2017; Solfrizzi et al., 2017; Livingston et al., 2020; El-Mezayen et al., 2022; Stach et al., 2021). The functional impact of micronutrient imbalance depends not only on serum concentrations but also on age, sex, genetic background, chronic inflammatory status, and lifestyle, so that individuals with similar laboratory profiles may display very different clinical trajectories (Ames, 2018; DeLuca et al., 2013; Harrison and May, 2009; Kennedy, 2016; Verdin, 2015; Etgen et al., 2012; Lloret et al., 2021; Li et al., 2017; Barbagallo et al., 2021; Cardoso et al., 2015; Livingston et al., 2020; Gasperi et al., 2019; Cater et al., 2024; Vinceti et al., 2018; Matusheski et al., 2021; Cortés-Albornoz et al., 2021; Bekdash, 2024; Badaeva et al., 2023; Figueroa-Méndez and Rivas-Arancibia, 2015). Mendelian randomization studies have begun to point toward potentially causal effects of circulating levels of specific vitamins and minerals such as vitamin E, B-vitamin status, and selenium on selected cognitive domains, although effect sizes are generally modest and clearly nutrient-specific (Zhang et al., 2023; Sano et al., 1997; Li et al., 2017; Cardoso et al., 2015; Wang et al., 2022; Badaeva et al., 2023). Physical activity consistently emerges as a key modifier: exercise-induced neurotrophin release, together with improved cerebral perfusion, appears to potentiate the positive impact of an adequate micronutrient status on neuroplasticity (Verdin, 2015; Solfrizzi et al., 2011; Solfrizzi et al., 2017; Livingston et al., 2020; Gasperi et al., 2019; Cater et al., 2024; Matusheski et al., 2021; Cortés-Albornoz et al., 2021; Bekdash, 2024; Mirarchi et al., 2023; Stach et al., 2021). From a preventive standpoint, personalized nutritional strategies grounded in biochemical monitoring, dietary assessment, and at least a basic neuropsychological profile, represent a pragmatic way to support cognitive health, particularly in vulnerable populations Table 3. (Gombart et al., 2020; DeLuca et al., 2013; Kennedy, 2016; Zimmermann, 2009; Zhang et al., 2023; Balion et al., 2012; Hisada et al., 2022; Bailey et al., 2015; Mayo-Wilson et al., 2011; He et al., 2014; Bjelakovic et al., 2007; Troesch et al., 2015; Mikkelsen et al., 2017; Etgen et al., 2012; Travica et al., 2017; Smith et al., 2010; Ford and Almeida, 2019; Velasco et al., 2018; Bougma et al., 2013; Sano et al., 1997; Lloret et al., 2021; Bozonet and Carr, 2019; Hare and Double, 2016; Li et al., 2017; Cardoso et al., 2015; Solfrizzi et al., 2011; Wu and Sun, 2017; Solfrizzi et al., 2017; Livingston et al., 2020; Wang et al., 2022; Vinceti et al., 2018; Bekdash, 2024; Badaeva et al., 2023; El-Mezayen et al., 2022; Pinto et al., 2020; Bath, 2024). This approach may be especially relevant for older adults, pregnant women, and individuals with restrictive eating patterns or a high prevalence of subclinical deficiencies, as is the case in several Latin American settings (Zimmermann, 2009; Hisada et al., 2022; Derbyshire, 2018; Bailey et al., 2015; Mayo-Wilson et al., 2011; Troesch et al., 2015; Velasco et al., 2018; Bougma et al., 2013; Lozoff and Georgieff, 2006; Hare and Double, 2016; Solfrizzi et al., 2011; Wu and Sun, 2017; Solfrizzi et al., 2017; Pinto et al., 2020; Bath, 2024). Regional data indicate that combined micronutrient supplementation, embedded within broader community programs, can be a realistic component of strategies to preserve cognitive function in nutritionally vulnerable older populations, particularly when accompanied by education and moderate physical activity (Balion et al., 2012; Troesch et al., 2015; Solfrizzi et al., 2011; Wu and Sun, 2017; Solfrizzi et al., 2017; Bekdash, 2024; El-Mezayen et al., 2022; Stach et al., 2021).

**TABLE 3:** Life-course determinants of micronutrient deficiency, key nutrients, biological mechanisms, and brain-related outcomes.

Life stage	Drivers of micronutrient deficiency	Key micronutrients for brain health	Main biological mechanisms	Brain-related consequences	Key references
First 1,000 days (conception–2 years)	Maternal undernutrition, low dietary diversity, suboptimal breastfeeding, infection-related nutrient losses	Iron, iodine, zinc, folate, vitamin B12, DHA	Neurogenesis, synaptogenesis, myelination, neurotransmitter synthesis	Impaired neurodevelopment, delayed cognitive milestones, reduced IQ, behavioral dysregulation	Prado and Dewey (2014), Black et al. (2017), Georgieff (2020), Cusick and Georgieff (2016), Nyaradi et al. (2013)
Childhood and adolescence	Hidden hunger, selective eating patterns, diets high in ultra-processed foods	Iron, zinc, vitamin B12, iodine, omega-3 fatty acids	Synaptic plasticity, neuronal signaling, neurotransmitter regulation, energy metabolism	Attention deficits, learning difficulties, poorer academic performance, emotional dysregulation	Bourre (2006), Nyaradi et al. (2013), Best et al. (2010), Grantham-McGregor et al. (2007)
Adulthood	Restrictive or unbalanced diets, chronic disease, gastrointestinal malabsorption, medication–nutrient interactions	B-vitamins (B6, B9, B12), magnesium, iron, vitamin D, omega-3 fatty acids	Homocysteine metabolism, mitochondrial energy production, neurotransmitter synthesis, oxidative stress regulation	Memory complaints, reduced executive function, fatigue, depression and anxiety symptoms	(Kennedy, 2016; Verdin, 2015; Mikkelsen et al., 2016; O’Leary and Samman, 2010)
Older age	Reduced appetite, dentition problems, social isolation, polypharmacy, reduced vitamin D synthesis	Vitamin B12, folate, vitamin D, calcium, antioxidants (vitamins C and E), omega-3 fatty acids	Neuroprotection, anti-inflammatory pathways, antioxidant defense, maintenance of neuronal membrane integrity	Accelerated cognitive decline, increased dementia risk, late-life depression, impaired functional autonomy	Verdin (2015), Mikkelsen et al. (2016), Roberts et al. (2019)

In therapeutic contexts, trials using single micronutrients have produced heterogeneous results, but certain patterns are emerging: benefits tend to concentrate in individuals with documented deficiency, at early stages of cognitive decline, and when supplementation is combined with lifestyle modification (DeLuca et al., 2013; Kennedy, 2016; Verdin, 2015; Balion et al., 2012; Bjelakovic et al., 2007; Etgen et al., 2012; Travica et al., 2017; Smith et al., 2010; Ford and Almeida, 2019; Sano et al., 1997; Lloret et al., 2021; Cardoso et al., 2015; Solfrizzi et al., 2011; Wu and Sun, 2017; Solfrizzi et al., 2017; Livingston et al., 2020; Wang et al., 2022; Gasperi et al., 2019; Cater et al., 2024; Vinceti et al., 2018; Bekdash, 2024; Badaeva et al., 2023; Mirarchi et al., 2023; MaretG, 2024; Figueroa-Méndez and Rivas-Arancibia, 2015; Stach et al., 2021). For instance, interventions that combine aerobic–resistance exercise, cognitive training, and vitamin D supplementation have yielded greater cognitive improvements than any of these strategies alone, supporting a multidomain intervention model (Verdin, 2015; Lloret et al., 2021; Solfrizzi et al., 2011; Solfrizzi et al., 2011; Livingston et al., 2020; Cater et al., 2024; Matusheski et al., 2021; Cortés-Albornoz et al., 2021; Bekdash, 2024; Mirarchi et al., 2023). Similarly, programs that integrate nutrition education, dietary optimization, and regular physical activity in community-dwelling older adults without dementia have been able to maintain or even enhance performance in memory and executive function tasks over short- and medium-term follow-up (Balion et al., 2012; Troesch et al., 2015; Solfrizzi et al., 2011; Wu and Sun, 2017; Solfrizzi et al., 2017; Bekdash, 2024; El-Mezayen et al., 2022; Stach et al., 2021) Table 4.

**TABLE 4:** Gene–micronutrient interactions influencing brain function and disease susceptibility.

Gene/molecular target	Micronutrient interaction	Brain-related consequences	Clinical implications	Key references
VDR (Vitamin D Receptor)	Vitamin D	Regulation of neurotrophic signaling pathways (including BDNF–TrkB and CREB), modulation of neuroimmune responses, redox homeostasis, and maintenance of synaptic stability in hippocampal and cortical circuits	VDR polymorphisms contribute to inter-individual variability in cognitive performance, mood regulation, and responsiveness to vitamin D supplementation across the lifespan	(DeLuca et al., 2013; Balion et al., 2012; Etgen et al., 2012; Mikkelsen et al., 2016; Black et al., 2017)
APOE (ε4 and related variants)	Iron; Vitamins C and E	Enhanced oxidative stress, altered lipid peroxidation handling, and regional iron accumulation in hippocampus and cortex, increasing vulnerability to synaptic dysfunction and neurodegeneration	Modifies risk, age of onset, and progression of Alzheimer’s disease; may influence therapeutic responses to antioxidant strategies and iron-modulating interventions	(Harrison and May, 2009; Lozoff and Georgieff, 2006; Beard, 2003; Lane et al., 2018; Hare and Double, 2016; Das et al., 2021; Cusick and Georgieff, 2016; Nyaradi et al., 2013; Best et al., 2010; Grantham-McGregor et al., 2007; daCunha Germano et al., 2023; La Torre et al., 2023; Icer et al., 2021)
MTHFR (C677T polymorphism)	Folate (Vitamin B9), Vitamin B12 (and indirectly B6 via homocysteine metabolism)	Reduced methylation capacity and elevated homocysteine levels; impaired DNA and histone methylation; effects on white matter integrity, vascular health, and cognitive function	Determines susceptibility to cognitive decline, depression, and vascular cognitive impairment; modulates the efficacy of B-vitamin supplementation in lowering homocysteine and slowing brain atrophy	(Kennedy, 2016; Smith et al., 2010; Ford and Almeida, 2019; Wang et al., 2022; Cortés-Albornoz et al., 2021; Kaye et al., 2025a)
Zinc-Finger Transcription Factors	Zinc	Altered transcriptional control of genes involved in synaptic plasticity, antioxidant defense, neurodevelopment, and cellular stress responses	Zinc deficiency or dysregulation may disproportionately affect neuronal resilience, learning capacity, and adaptive plasticity, particularly during aging and metabolic stress	(Gómez-Pinilla, 2008; Zhang et al., 2023; Li et al., 2017; Solfrizzi et al., 2011; Livingston et al., 2020)
Thyroid Hormone Transporters and Deiodinases (MCT8, DIO2, DIO3)	Iodine	Region- and stage-specific regulation of thyroid hormone availability during brain development, with differential effects on cortical and cerebellar maturation, neuronal migration, and myelination	Provides a mechanistic basis for brain-region- and developmental-stage–specific cognitive effects of iodine deficiency or excess, informing public health and clinical strategies	(Zimmermann, 2009; Hisada et al., 2022; Velasco et al., 2018; Bougma et al., 2013; Diachenko et al., 2024; AlBlooshi, 2025)

Where possible, we indicate whether findings derive from habitual dietary intake, food based fortification, or pharmacological supplementation, as these modalities differ in dose, bioavailability, and the context in wich deficiencies arise (Bourre, 2006; Kennedy, 2016; Balion et al., 2012; Jia et al., 2019; AlBlooshi, 2025; Stach et al., 2021; Liwinski and Lang, 2023; Kaye et al., 2025b).

### Balancing benefits and risks: controversies and trade-offs in micronutrient strategies for brain health

Micronutrient-based strategies for brain health are not free of trade-offs, and recent data urge a more cautious, individualized view. High-dose antioxidant supplementation, particularly vitamin E, illustrates this tension: while physiological levels may support neuroprotection, meta-analytic and clinical analyses suggest that high dosages can worsen outcomes in some groups, including an increased risk of all-cause mortality and inconsistent benefits in Alzheimer’s disease and related conditions (Bjelakovic et al., 2007; Icer et al., 2021; Kaye et al., 2025a). Iron offers a different but equally complex example. Mendelian randomization and imaging–genetic studies now indicate that both systemic iron status and region-specific iron accumulation in subcortical nuclei can causally influence the risk of major depression, schizophrenia, and other psychiatric disorders, implying that “normalizing” iron without considering genetic background and brain distribution may be counterproductive in certain individuals (Lane et al., 2018; Hare and Double, 2016; Das et al., 2021). At the same time, work on neuroinflammation and microglial biology highlights that the impact of micronutrients on brain immune tone is highly context-dependent, shaped by broader dietary patterns, gut–brain signaling, and disease stage (Matusheski et al., 2021). Even for combinations that appear promising—such as structured physical activity plus multimicronutrient supplementation to support cognition—the available randomized trials and meta-analyses show substantial heterogeneity in populations, doses, and outcomes, reminding us that mechanistic plausibility does not automatically translate into uniform clinical benefit (Bjelakovic et al., 2007; Troesch et al., 2015; Solfrizzi et al., 2011; Wu and Sun, 2017; Solfrizzi et al., 2017; Icer et al., 2021).

## Discussion and conclusion

Overall, the evidence reviewed supports the notion that brain health depends on a finely tuned micronutrient balance, in which both insufficiency and excess may perturb neural homeostasis (Bourre, 2006; Prado and Dewey, 2014; Gómez-Pinilla, 2008; Ames, 2018; DeLuca et al., 2013; Brigelius-Flohé and Traber, 1999; Harrison and May, 2009; Kennedy, 2016; Verdin, 2015; Zimmermann, 2009; Zhang et al., 2023; Balion et al., 2012; Bjelakovic et al., 2007; Etgen et al., 2012; Smith et al., 2010; Ford and Almeida, 2019; Lozoff and Georgieff, 2006; Beard, 2003; Lane et al., 2018; Lloret et al., 2021; Hare and Double, 2016; Das et al., 2021; Li et al., 2017; Barbagallo et al., 2021; Cardoso et al., 2015; Solfrizzi et al., 2011; Wu and Sun, 2017; Solfrizzi et al., 2017; Livingston et al., 2020; Wang et al., 2022; Gasperi et al., 2019; Cater et al., 2024; Badaeva et al., 2023; MaretG, 2024; Figueroa-Méndez and Rivas-Arancibia, 2015). Vitamins and minerals influence neurotransmission, synaptic remodeling, cellular energy metabolism, and neuronal structural integrity through tightly interconnected molecular and physiological pathways (Bourre, 2006; Prado and Dewey, 2014; Gómez-Pinilla, 2008; Ames, 2018; DeLuca et al., 2013; Verdin, 2015; Zimmermann, 2009; Zhang et al., 2023; Balion et al., 2012; Etgen et al., 2012; Travica et al., 2017; Maden, 2007; Lozoff and Georgieff, 2006; Beard, 2003; Sano et al., 1997; Lloret et al., 2021; Hare and Double, 2016; Das et al., 2021; Barbagallo et al., 2021; Cardoso et al., 2015; Roberts et al., 2019; Jia et al., 2019; Gasperi et al., 2019; Bekdash, 2024; Badaeva et al., 2023; Georgieff, 2020; Cusick and Georgieff, 2016; Best et al., 2010; Olson and Mello, 2010; Hirota, 2023; daCunha Germano et al., 2023; La Torre et al., 2023; MaretG, 2024; Popa et al., 2021). When these systems are disturbed, even subtly functional consequences may arise that affect cognition, emotional regulation, and behavior at different stages of life, often before fully developed clinical syndromes are recognized (Prado and Dewey, 2014; Gómez-Pinilla, 2008; Ames, 2018; Kennedy, 2016; Zimmermann, 2009; Balion et al., 2012; Hisada et al., 2022; Derbyshire, 2018; Bailey et al., 2015; He et al., 2014; Troesch et al., 2015; Mikkelsen et al., 2017; Etgen et al., 2012; Presse et al., 2008; Travica et al., 2017; Smith et al., 2010; Ford and Almeida, 2019; Lozoff and Georgieff, 2006; Hare and Double, 2016; Das et al., 2021; Li et al., 2017; Cardoso et al., 2015; Solfrizzi et al., 2011; Wu and Sun, 2017; Solfrizzi et al., 2017; Livingston et al., 2020; Wang et al., 2022; Vinceti et al., 2018; Badaeva et al., 2023; Emekli-Alturfan and Alturfan, 2023; AlBlooshi, 2025; Figueroa-Méndez and Rivas-Arancibia, 2015; Bath, 2024).

From a clinical perspective, these findings argue against relying solely on isolated biochemical measurements or purely symptom-based approaches (Ames, 2018; Kennedy, 2016; Zimmermann, 2009; Zhang et al., 2023; Balion et al., 2012; Hisada et al., 2022; Travica et al., 2017; Smith et al., 2010; Ford and Almeida, 2019; Velasco et al., 2018; Bougma et al., 2013; Lozoff and Georgieff, 2006; Beard, 2003; Hare and Double, 2016; Das et al., 2021; Li et al., 2017; Cardoso et al., 2015; Solfrizzi et al., 2011; Wu and Sun, 2017; Solfrizzi et al., 2017; Livingston et al., 2020; Wang et al., 2022; Vinceti et al., 2018; Badaeva et al., 2023; Emekli-Alturfan and Alturfan, 2023; AlBlooshi, 2025; Figueroa-Méndez and Rivas-Arancibia, 2015). Whenever appropriate, a comprehensive assessment of micronutrient status should be integrated with neuropsychological testing capable of capturing early changes in attention, memory, processing speed, executive function, and emotional control, thereby allowing a more nuanced interpretation of laboratory results (Zimmermann, 2009; Balion et al., 2012; Etgen et al., 2012; Presse et al., 2008; Travica et al., 2017; Ford and Almeida, 2019; Bozonet and Carr, 2019; Das et al., 2021; Solfrizzi et al., 2011; Wu and Sun, 2017; Solfrizzi et al., 2017; Livingston et al., 2020; Bekdash, 2024; Popa et al., 2021; Emekli-Alturfan and Alturfan, 2023; Figueroa-Méndez and Rivas-Arancibia, 2015; Stach et al., 2021). Implementing this approach opens the door to catching early signs of cognitive vulnerability and delivering interventions that are both timely and finely calibrated to individual needs, whether in clinic settings or at the population level (DeLuca et al., 2013; Zimmermann, 2009; Zhang et al., 2023; Balion et al., 2012; Hisada et al., 2022; Derbyshire, 2018; Troesch et al., 2015; Mikkelsen et al., 2017; Etgen et al., 2012; Travica et al., 2017; Ford and Almeida, 2019; Velasco et al., 2018; Bougma et al., 2013; Lloret et al., 2021; Bozonet and Carr, 2019; Hare and Double, 2016; Li et al., 2017; Cardoso et al., 2015; Solfrizzi et al., 2011; Wu and Sun, 2017; Solfrizzi et al., 2017; Livingston et al., 2020; Wang et al., 2022; Gasperi et al., 2019; Matusheski et al., 2021; Cortés-Albornoz et al., 2021; Grantham-McGregor et al., 2007; Icer et al., 2021; Kaye et al., 2025a; Popa et al., 2021; Diachenko et al., 2024; AlBlooshi, 2025).

A critical but often overlooked aspect of brain health lies in how we move our bodies in relation to what we eat (Ames, 2018; DeLuca et al., 2013; Kennedy, 2016; Verdin, 2015; Zhang et al., 2023; Balion et al., 2012; Etgen et al., 2012; Solfrizzi et al., 2011; Solfrizzi et al., 2017; Livingston et al., 2020; Gasperi et al., 2019; Cater et al., 2024; Matusheski et al., 2021; Cortés-Albornoz et al., 2021; Bekdash, 2024; Badaeva et al., 2023; Mirarchi et al., 2023; Stach et al., 2021). The research is increasingly clear: when people exercise regularly, combining cardiorespiratory and strength-building work, the positive effects of adequate micronutrient intake on the brain appear to be magnified, through pathways such as enhanced blood flow to brain tissue, released growth factors, and more efficient cellular energy metabolism (Verdin, 2015; Solfrizzi et al., 2011; Solfrizzi et al., 2017; Livingston et al., 2020; Gasperi et al., 2019; Cater et al., 2024; Matusheski et al., 2021; Cortés-Albornoz et al., 2021; Bekdash, 2024; Mirarchi et al., 2023; Stach et al., 2021). Seen this way, micronutrients become the raw materials that fuel the brain’s adaptive response to physical challenge, while exercise itself sharpens the brain’s ability to use those nutrients. This interplay proves particularly valuable as we age (Balion et al., 2012; Troesch et al., 2015; Solfrizzi et al., 2011; Wu and Sun, 2017; Solfrizzi et al., 2017; Livingston et al., 2020; Bekdash, 2024; Mirarchi et al., 2023; El-Mezayen et al., 2022; Stach et al., 2021).

Building a truly effective brain-health program thus requires careful measurement of both nutritional status and exercise capacity, smart correction of any nutritional shortfalls through diet or supplements, designing fitness routines that match each person’s abilities, and using repeated testing to track cognitive changes over time (Ames, 2018; DeLuca et al., 2013; Zimmermann, 2009; Zhang et al., 2023; Hisada et al., 2022; Derbyshire, 2018; Bjelakovic et al., 2007; Troesch et al., 2015; Etgen et al., 2012; Travica et al., 2017; Ford and Almeida, 2019; Velasco et al., 2018; Bougma et al., 2013; Lloret et al., 2021; Bozonet and Carr, 2019; Hare and Double, 2016; Li et al., 2017; Cardoso et al., 2015; Solfrizzi et al., 2011; Wu and Sun, 2017; Solfrizzi et al., 2017; Livingston et al., 2020; Wang et al., 2022; Vinceti et al., 2018; Bekdash, 2024; Badaeva et al., 2023; Icer et al., 2021; Emekli-Alturfan and Alturfan, 2023; AlBlooshi, 2025; Figueroa-Méndez and Rivas-Arancibia, 2015; El-Mezayen et al., 2022; Stach et al., 2021; Pinto et al., 2020; Bath, 2024). Not only can such an approach help maintain or improve thinking and mood in people who are currently well, but it also holds promise for preventing or slowing cognitive diseases, developmental delays, and depression (Ames, 2018; DeLuca et al., 2013; Kennedy, 2016; Zimmermann, 2009; Zhang et al., 2023; Balion et al., 2012; Etgen et al., 2012; Travica et al., 2017; Smith et al., 2010; Ford and Almeida, 2019; Lane et al., 2018; Bozonet and Carr, 2019; Das et al., 2021; Solfrizzi et al., 2011; Wu and Sun, 2017; Solfrizzi et al., 2017; Livingston et al., 2020; Wang et al., 2022; Cortés-Albornoz et al., 2021; Icer et al., 2021; Kaye et al., 2025a; Popa et al., 2021).

Today’s science in micronutrient research increasingly points to a simple but powerful fact: no two people respond to diet and exercise in quite the same way. Differences in our genes, the microorganism inhabiting the human gastrointestinal tract, the level of inflammation we carry, our body composition, and our daily habits all play a role in determining how well we benefit from good nutrition and physical activity (Ames, 2018; DeLuca et al., 2013; Kennedy, 2016; Verdin, 2015; Balion et al., 2012; Etgen et al., 2012; Lozoff and Georgieff, 2006; Lloret et al., 2021; Livingston et al., 2020; Gasperi et al., 2019; Cater et al., 2024; Matusheski et al., 2021; Cortés-Albornoz et al., 2021; Bekdash, 2024; Badaeva et al., 2023; Stach et al., 2021). This means that truly addressing brain health demands a team approach, bringing together researchers and clinicians who specialize in nutrition, brain biology, medicine, and behavior, while drawing on the growing toolkit of personalized medicine (Ames, 2018; Balion et al., 2012; Sano et al., 1997; Das et al., 2021; Solfrizzi et al., 2011; Wu and Sun, 2017; Solfrizzi et al., 2017; Livingston et al., 2020; Wang et al., 2022; Cortés-Albornoz et al., 2021; Best et al., 2010; Kaye et al., 2025a; Popa et al., 2021). The ultimate goal is to take what we now understand about how micronutrients work in the brain and turn that knowledge into practical, long-lasting approaches that keep our minds sharp and our emotions balanced throughout life (Gómez-Pinilla, 2008; Ames, 2018; DeLuca et al., 2013; Kennedy, 2016; Verdin, 2015; Zimmermann, 2009; Zhang et al., 2023; Balion et al., 2012; Troesch et al., 2015; Etgen et al., 2012; Smith et al., 2010; Ford and Almeida, 2019; Sano et al., 1997; Das et al., 2021; Solfrizzi et al., 2011; Wu and Sun, 2017; Solfrizzi et al., 2017; Livingston et al., 2020; Wang et al., 2022; Gasperi et al., 2019; Cater et al., 2024; Badaeva et al., 2023; MaretG, 2024; Stach et al., 2021).
